# Pretreatment naive T cells are associated with severe irAE following PD-1/CTLA-4 checkpoint blockade for melanoma

**DOI:** 10.1172/jci.insight.198203

**Published:** 2025-11-25

**Authors:** Kathryne E. Marks, Alice Horisberger, Mehreen Elahee, Ifeoluwakiisi A. Adejoorin, Nilasha Ghosh, Michael Postow, Laura Donlin, Anne R. Bass, Deepak A. Rao

**Affiliations:** 1 Division of Rheumatology, Inflammation, Immunity, Brigham and Women’s Hospital and Harvard Medical School, Boston, Massachusetts, USA.; 2 Division of Rheumatology, Hospital for Special Surgery and Weill Cornell Medicine, New York, New York, USA.; 3 Memorial Sloan Kettering Center and Weill Cornell Medical College, New York, New York, USA.; 4 HSS Research Institute, Hospital for Special Surgery, New York, New York, USA.

**Keywords:** Autoimmunity, Immunology, Oncology, Adaptive immunity, Cancer immunotherapy, T cells

## Abstract

Immune checkpoint inhibitors (ICIs) such as anti–PD-1 and anti–CTLA-4 antibodies are used to induce an immune response against many types of tumors. However, ICIs often also induce autoimmune responses, referred to as immune-related adverse events (irAEs), which occur unpredictably and at varying levels of severity. We utilized high-dimensional immunophenotyping of longitudinal blood samples from patients with metastatic melanoma treated with combination anti–PD-1/CTLA-4 therapy in a clinical trial to characterize alterations in immune profiles induced by combination ICI therapy and to identify immune features associated with severe irAE development. T cell profiling highlighted that ICI therapy induces prominent expansions of activated, CD38^hi^ CD4^+^ and CD8^+^ T cells, which are frequently bound by the therapeutic anti–PD-1 antibody, as well as substantial changes in Treg phenotypes. However, neither the baseline frequency nor the extent of expansion of these cell populations was associated with severe irAE development. Rather, naive CD4^+^ T cell abundance pretreatment was significantly associated with development of severe irAEs and with the number of irAEs developed. These results indicate the abundance of naive CD4^+^ T cells as a predictive feature for the development of severe irAEs following combination ICI therapy.

## Abstract 2

Immune checkpoint inhibitor therapies for cancer can induce unintended immune related adverse events (irAEs). Using mass cytometry immune profiling, this study identifies specific T cell features in pre-treatment blood samples from patients that are associated with subsequent development of irAEs after ICI therapy.

## Introduction

Immune checkpoint inhibitors (ICIs) have significantly advanced cancer treatment since their initial approval over a decade ago and contribute to lasting survival (1–3). ICI therapy with antibodies that block PD-1, PD-L1, CTLA-4, or LAG-3 have led to improved responses against cancers of skin, lung, kidney, and other tissues (4), and treatment with a combination of checkpoint blockade treatments can increase their efficacy (5). ICI therapies release T cells from inhibitory signals and activate them to recognize and kill tumor cells. However, ICI therapies can also cause systemic changes in T cell function, in addition to their intended effects on tumor-infiltrating T cells. Changes to circulating T cell populations following a combination of ICI therapies, such as anti–PD-1 together with anti–CTLA-4, can be distinct from treatment with either monotherapy (6–8). The extent of changes to well-defined subsets of circulating immune cell populations following combination PD-1/CTLA-4 blockade remains incompletely defined.

Systemic or extratumor T cell activation by ICI therapies can induce immune-related adverse events (irAEs) (9, 10). irAEs range in severity, timing, and organ system affected but are a frequent result of ICI therapies, occurring in up to 90% of people receiving combination anti–PD-1/CTLA-4 blockade therapy (11, 12). More than half of irAEs resulting from this combination are grade 3 or 4 and can necessitate hospitalization and cessation of therapy (11). Common irAEs include colitis, rashes, hepatitis, and arthritis among others (10, 13). While myositis and myocarditis are rarer irAEs, they are often severe and can result in mortality (10).

Specific clinical factors have been associated with increased risk of irAE or more severe irAEs in some studies, including high BMI, smoking, and male sex; however, these associations have not been consistently observed (14–17). Younger age has also been associated with more severe irAEs in some but not all studies (18, 19). Identification of cells in circulation that are associated with type or severity of irAE before or early in treatment would enable better prediction of irAE development. Studies of cellular profiles have yielded varied results regarding associations of T cell phenotypes associated with irAEs (20–22). CD4^+^ T cells, particularly memory CD4^+^ T cells and more TCR diversity, were reported in an observational study as associated with future irAE severity in a cohort of both anti–PD-1 alone and combination therapy–treated patients (22). However, a separate observational study found that naive CD4^+^ T cells were present at higher levels in patients with higher severity irAEs following either anti–PD-1, anti–CTLA-4, or combination anti-CTLA-4/anti–PD-1 therapy (21). Another observational study utilizing flow cytometry found no differences in naive CD4^+^ or CD8^+^ T cells in patients treated with ICI therapy with an irAE versus without any irAEs (20); thus, substantial uncertainty remains about T cell phenotypes predictive of irAE development.

Utilizing PBMCs collected from well-characterized patients through the Adaptively Dosed ImmunoTherapy Trial (ADAPT-IT) (5), we aimed to determine the specific effects of anti–PD-1/CTLA-4 combination therapy on immune cell populations in blood and to determine associations of blood immune cell features with irAE severity in the setting of this clinical trial. Previous analysis of patients in the ADAPT-IT revealed an increase in proliferating T cells after 1 dose of combination therapy. Here, we extend the analysis of patients in this trial by using high-dimensional mass cytometry profiling of circulating T cells to identify cellular features in the baseline, pretreatment samples associated with irAE development (23). We show dramatic expansion of activated and proliferating T cells that are directly acted upon by anti–PD-1 therapy. Notably, this dramatic cellular activation is not associated with development of irAEs. Rather, our broad immune profiling approach demonstrated that the level of naive T cells, especially naive CD4^+^ T cells, before ICI therapy is significantly associated with development of severe irAEs following combination anti–PD-1/CTLA-4 therapy in this clinical trial setting.

## Results

### Patient characteristics.

We obtained PBMCs from patients with advanced melanoma enrolled in the ADAPT-IT (5). Patients in this multicenter, single-arm, phase II clinical trial were treated with 2 doses of both nivolumab (1 mg/kg), an anti–PD-1 therapy, and ipilimumab (3 mg/kg), an anti–CTLA-4 therapy, either followed by nivolumab monotherapy every 2 or 4 weeks or 2 more doses of combination therapy followed by nivolumab monotherapy every 2 or 4 weeks. Patients in this trial had no prior exposure to ICI-therapy and no active autoimmune disease at the time of participation. The demographics of analyzed patients are summarized in Table 1 and Supplemental Table 1 (supplemental material available online with this article; https://doi.org/10.1172/jci.insight.198203DS1). Among the overall cohort, the mean age was 60 years, and the cohort was 59% male.

After treatment initiation, participants were diagnosed with irAEs of varying severity, type, and onset time. The most common irAEs were gastrointestinal and endocrine related. The median number of irAEs per participant was 3. In total, 61% of paired patients had already been diagnosed with at least 1 irAE at the 6-week collection time point, and the majority of participants (95%) were diagnosed with an irAE at some point during the trial. For analysis of baseline sample associations with irAE severity, we considered all irAEs diagnosed at any time following the initial dosing. irAEs were graded 0–5 using the Common Terminology Criteria for Adverse Events (CTCAE) and were divided into low (grade 1–2) and high (grade 3–5) severity (24).

**Figure 1:**
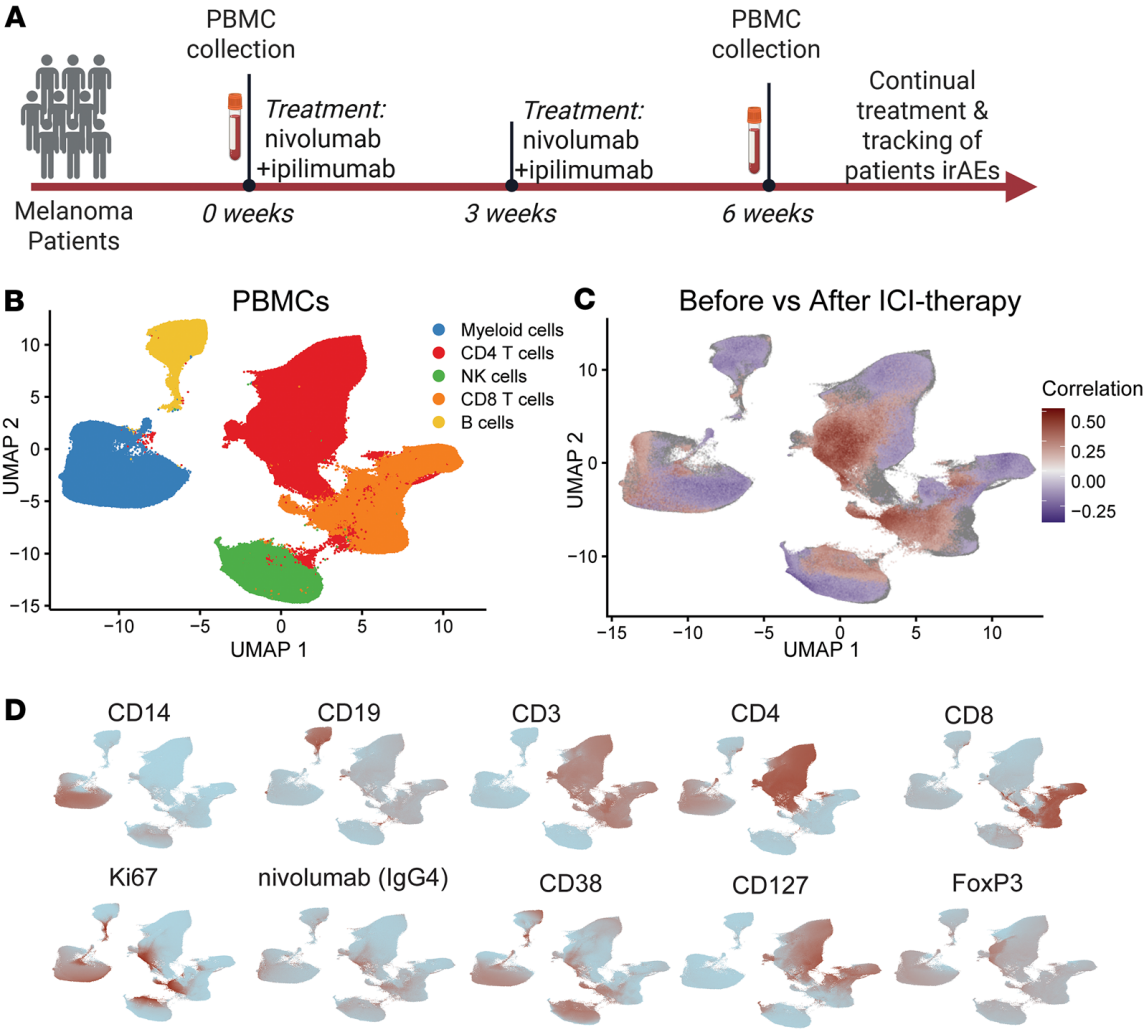
Changes in circulating cell frequency following combination ICI therapy. (**A**) Schematic of experimental setup of analysis of PBMCs from patients with melanoma undergoing ICI treatment. (**B**) UMAP representation following unsupervised clustering of PBMCs of melanoma patients before and after treatment (*n* = 49 patients; 31 paired). (**C**) CNA of before treatment samples versus after treatment samples. Red cells indicate neighborhoods significantly associated with posttreatment samples, while blue cells indicate neighborhoods significantly associated with pretreatment samples. CNA (28) global *P* = 0.023; FDR < 0.1. (**D**) Feature plots of the indicated markers on the PBMC UMAP. Red indicates high expression.

We obtained PBMCs before treatment from 49 unique patients and paired blood 6 weeks after treatment initiation (after 2 doses of combination ICI therapy) from 31 of these patients. Cryopreserved PBMCs were processed for mass cytometry using a panel of antibodies to identify major lineages as well as specific T cell phenotypes (Figure 1A). Gated live cells, gated using FlowJo, were corrected for batch effects using the CyCombine R package (25) and then clustered in an unsupervised manner using Seurat (26). We then analyzed the data for effects of therapy and associations with irAE severity.

### Changes in proportions of immune cells following combination ICI therapy.

We looked broadly at changes in frequencies of circulating immune cell populations induced by ICI therapy. The total proportions of CD4^+^ T cells, CD8^+^ T cells, NK cells, or myeloid cells did not consistently change following initiation of combination ICI therapy (Figure 1B and Supplemental Figure 1, A and B). The frequency of total circulating B cells, however, was reduced following ICI therapy (Supplemental Figure 1A). To gain further insight into specific B cell changes following checkpoint blockade and to determine any association with irAE severity in this cohort, we performed flow cytometry focused on B cell populations on the same samples. We confirmed a reduction of CD19^+^ cells in PBMCs in this dataset (Supplemental Figure 2A). However, among CD19^+^ B cells, we observed increases in the proportion of both CD21lo B cells and CD38^hi^CD27^hi^ plasmablasts (Supplemental Figure 2, B and C). The frequency of CD27^+^ memory B cells did not change after therapy; however, IgG^+^ memory B cells decreased significantly in most patients (Supplemental Figure 2, D and E). Despite the changes in B cell frequencies, the baseline frequencies of each B cell population and the changes induced by ICI therapy in each B cell population were similar between those who were diagnosed with a severe irAE or a mild irAE (Supplemental Figure 3, A–D).

In addition to B cells, we also analyzed frequencies of plasmacytoid DCs (pDCs) before and after combination ICI therapy by flow cytometry due to their association with autoimmune diseases and their production of type I IFN, a factor that promotes expansion of CD38^hi^CD8^+^ T cells in ICI-associated arthritis (27). pDCs, identified as CD3^–^CD19^–^CD14^–^CD11C^–^BDCA2^+^CD123^+^ cells, were decreased following ICI therapy, with similar decreases in patients with and without severe irAE development (Supplemental Figure 2, F and L). The frequency of pDCs at baseline was not associated with the irAE severity, and despite decreasing in most patients, the level of decrease in pDC frequency did not vary with irAE severity level (Supplemental Figure 2M).

To assess effects of ICI therapy on circulating immune cell phenotypes broadly yet with high resolution, we utilized covarying neighborhood analysis (CNA) within the mass cytometry data (28). We observed significant differences in T cells, including clear increases in the posttreatment group in subsets of CD4^+^ and CD8^+^ T cells (Figure 1C). Expanded T cells had phenotypes of memory T cell subsets characterized by high levels of CD38 and low levels of CD127, and they express the proliferation marker Ki67 (Figure 1D).

### Changes in circulating T cells following combination ICI therapy.

**Figure 2:**
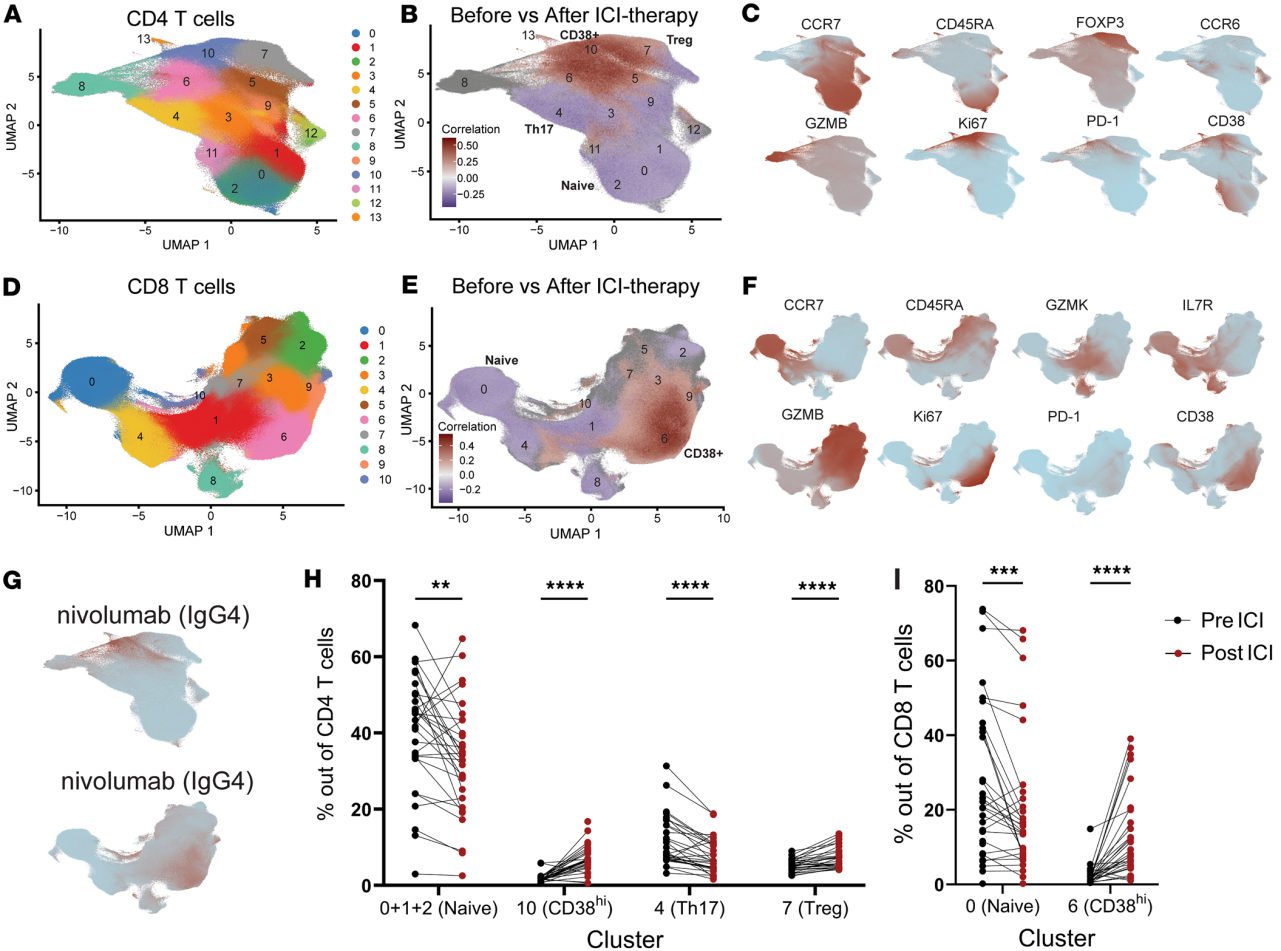
Changes in T cells following combination ICI therapy. (**A**) UMAP representation following unsupervised clustering of CD4 T cells. (**B**) CNA of pretreatment samples versus after treatment samples. CNA (28) global *P* = 0.001; FDR <0.1. (**C**) Feature plots of the indicated markers on the CD4 T cell UMAP. (**D**) UMAP representation following unsupervised clustering of CD8 T cells. (**E**) CNA of before treatment samples versus after treatment samples. *P* = 0.001. (**F**) Feature plots of the indicated markers on the CD8 T cell UMAP. (**G**) Feature plots of in the CD4 (left) and CD8 (right) T cells indicating detection of nivolumab. (**H** and **I**) Paired analysis of percent of indicated cluster in CD4 or CD8 T cells. **P < 0.01, ***P < 0.001, ****P < 0.0001 by Wilcoxon paired test.

To evaluate changes in T cells more granularly, we performed focused analyses on CD4^+^ T cells and CD8^+^ T cells (Figure 2, A and D, and Supplemental Figure 3, A and B). CNA and cluster analyses demonstrated widespread changes in both CD4^+^ and CD8^+^ T cell composition following ICI therapy (Figure 2, B and E). The most clearly expanded CD4^+^ T cells were in CD4 Cluster 10, which showed high levels of CD38 and low levels of CD127 as well as expression of granzyme B, granzyme K, perforin, and HLA-DR (Figure 2, C and H, and Supplemental Figure 3A). These CD4^+^ T cells were the highest expressors of the proliferation marker Ki67 (Figure 2C). Similarly, CD8 Cluster 6, which closely resembles CD4 Cluster 10, was dramatically induced following ICI therapy (Figure 2, F and I), consistent with prior analyses (27). Interestingly, CD4 Cluster 8, which was characterized by granzyme B expression, but not CD38, did not change following ICI therapy (Figure 2, B and C, and Supplemental Figure 3C). Thus, cytotoxic CD4^+^ T cells in general did not increase, but rather a separate population of proliferating CD4s with granzyme B expression increased. We confirmed these changes to T cell populations using biaxial gating (Supplemental Figure 3, E and F).

Our mass cytometry panel included an antibody against IgG4, the isotype of nivolumab (29), enabling detection and evaluation of cells bound by nivolumab. Most of the IgG4^+^ cells, the cells currently being treated by nivolumab, were identified in the expanded Ki67^hi^CD38^hi^ cluster. This observation was found in both the CD4 and CD8^+^ T cells (Figure 2G). Both CD8^+^ and CD4^+^ T cells were bound by nivolumab, although CD8^+^ T cells had a higher mean binding of nivolumab (Supplemental Figure 5, A and B). The frequency of nivolumab-bound CD4^+^ or CD8^+^ T cells was not higher in those patients with current or future severe irAEs (grade 3–4) compared with patients with less severe irAEs (grade 1–2) (Supplemental Figure 5C). Similarly, the expression of PD-1 on T cells at baseline was not different between patients with irAEs of grades 0–2 irAEs (Low Severity) and grades 3–4 irAEs (High Severity) and was not correlated with time of irAE onset or the total number of irAEs (Supplemental Figure 5D). Other CD4^+^ T cell cluster changes included a decrease in the proportion of Cluster 4, characterized by CCR6 and CD161, representing a decrease in Th17 cells (Figure 2H and Supplemental Figure 3, C–F). Additionally, both naive CD4^+^ and CD8^+^ T cells exhibited a slight but significant decrease in proportion following ICI therapy (Figure 2, H and I, and Supplemental Figure 3, C–F).

We also observed a significant increase in the frequency of Cluster 7 following combination ICI therapy. Cluster 7 was characterized by FOXP3, CD25, and Helios, consistent with CD4^+^ Tregs (Figure 2H). In this cohort, ICI therapy induced an overall increase in frequency of CD25^hi^CD127^–^CD4^+^ T cells (Supplemental Figure 4, A and C). Within Tregs, we observed clear changes in Treg phenotypes following ICI therapy (Supplemental Figure 4B). Several markers related to Treg function and activation increased, including FOXP3, Helios, CD39, CD38, ICOS, HLA-DR, and Ki67, while TCF1 decreased (Supplemental Figure 4, D and E).

### Frequency of naive T cells is associated with irAE severity.

**Figure 3:**
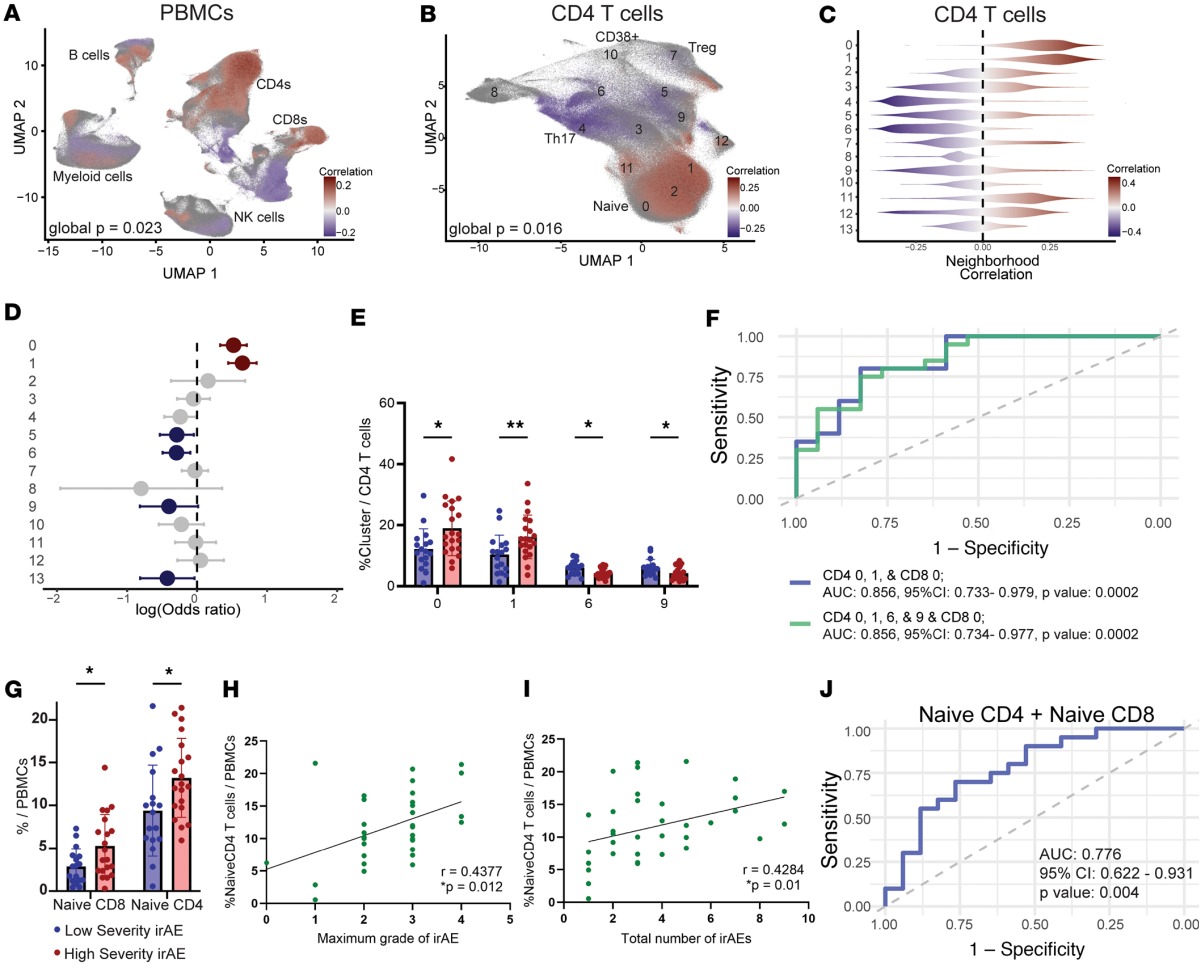
Naive T cell levels before ICI therapy are associated with irAE severity. (**A** and **B**) CNA for high severity irAE PBMCs or CD4 T cells before ICI treatment. Red correlation values indicate significant positive association with development of severe irAE. All colored cells are filtered for FDR < 0.1. (**C**) Correlation values of cells shown in **B** sorted by cluster membership of the correlated cells. Red indicates positive correlation with severe irAEs. (**D**) MASC analysis of CD4 T cells. Red indicates positive association with high severity irAE, and blue indicates association with low severity irAE. *P* < 0.05 and are filtered for FDR < 0.1. Bars indicate confidence interval. (**E**) Proportion of CD4 clusters at baseline with *P* < 0.05 for comparison of low versus high severity. (**F**) ROC curves of proportion of CD4 clusters 0, 1, and CD8 Cluster 0 (in blue) and CD4 clusters 0, 1, 6, and 9 and CD8 Cluster 0 (in green) for development of severe irAE. (**G**) Gating of mass cytometry data to quantify frequency of naive T cells (CD3^+^CD4^+^CD45RA^+^CCR7^–^ or CD3^+^CD8^+^CD45RA^+^CCR7^–^) out of total PBMCs of patients grouped by irAE severity. *P < 0.05 by Mann-Whitney *U* test. (**H** and **I**) Spearman correlation of gated naive CD4 T cell proportion before ICI treatment with maximum grade or total number of irAEs (*n* = 37 patients). (**J**) ROC curve of proportion of naive CD4 T cells and naive CD8 T cells for development of severe irAE.

We next attempted to identify PBMC populations in the baseline, pretreatment samples that were associated with severity of future irAE following ICI therapy. We first employed CNA to look for correlations of fine-grained neighborhoods with severity of subsequent irAEs (28). CNA comparing PBMCs from patients who subsequently developed high grade irAEs (High Severity) to those who did not (Low Severity) identified several cell neighborhoods significantly correlated with irAE severity (Figure 3A). The cell neighborhoods with positive correlation values correlated with development of severe irAEs; these neighborhoods were primarily composed of naive CD4^+^ and naive CD8^+^ T cells (Figure 3A). Among CD4^+^ T cells, the association of naive CD4^+^ T cells with development of severe irAEs remained even when adjusting for age and sex (Figure 3, B and C). To confirm this association using a cell cluster–based analysis, we implemented mixed-effects modeling of association of single cells (MASC), which identifies cell clusters associated with a clinical variable using a mixed-effect logistic regression model at a single-cell level. Consistent with CNA results, MASC analyses controlling for age and sex also identified CD4 Clusters 0 and 1 as significantly associated with severe irAEs, while CD4 Clusters 5, 6, 9, and 13 were negatively associated with severe irAEs (Figure 3D). CD4 Clusters 0 and 1 contained naive CD4 T cells, while Clusters 5, 6, 9, and 13 contained memory CD4 populations including those expressing CD40L, Tbet, CD57, and granzyme B. Among CD8^+^ T cell clusters, MASC analysis highlighted the naive CD8^+^ T cell cluster, Cluster 0, as associated with development of severe irAEs; however, this observation did not pass correction for multiple testing (Supplemental Figure 6E).

The proportions of CD4^+^ cluster T cell clusters 0 and 1, out of all CD4 T cells, were significantly higher at baseline in those who developed high severity irAEs, and the proportions of clusters 6 and 9 were significantly lower (Figure 3E). Additionally, the proportion of CD8 Cluster 0 out of all CD8 T cells was significantly higher in those who developed high severity irAEs than those with low severity irAEs (Supplemental Figure 6F). In ROC analysis, the AUC for the baseline proportion of CD4 T cell clusters 0, 1, 6, 9, and CD8 Cluster 0 could predict severe irAE (AUC 0.856; 95% CI, 0.734–0.934) (Figure 3F). The same analysis including naive T cells only, CD4 T cell clusters 0 and 1, and CD8 Cluster 0, resulted in the same AUC (Figure 3F).

We next attempted to confirm these observations using biaxial gating of the mass cytometry data. Biaxial gating of the memory CD4 T cell subsets could not replicate the clusters associated with lower severity irAEs (clusters 6 and 9); therefore, we did not pursue these further. However, naive CD4^+^ or CD8^+^ T cells, corresponding to clusters 0 and 1, could be easily gated as CD19^–^CD14^–^CD3^+^CD4^+^/CD8^+^CD45RA^+^CCR7^+^. Patients who developed severe irAEs had a higher proportion of gated naive CD4 and CD8^+^ T cells out of all PBMCs at baseline than patients who did not develop severe irAEs (Figure 3G). Furthermore, the frequency of all naive CD4^+^ T cells (*r* = 0.4377, *P* = 0.012) and naive CD8^+^ T cells (*r* = 0.3854, *P* = 0.029) before therapy was significantly correlated with maximum grade of future irAE (Figure 3H and Supplemental Figure 6C). Frequency of naive CD4^+^ T cells, but not naive CD8^+^ T cells, at baseline was also correlated with the total number of irAEs per patient (*r* = 0.4284, *P* = 0.01) (Figure 3I and Supplemental Figure 6D). ROC analysis of gated naive CD4 and CD8^+^ T cells predicted onset of severe irAE (AUC 0.776; 95% CI, 0.622–0.931) (Figure 3J). Naive CD4 T cell proportion at baseline was not associated with overall survival of melanoma within this cohort (Supplemental Figure 6, F and G) (30).

## Discussion

In this study, we utilized high-dimensional mass cytometry analyses to assess both changes in circulating immune cell profiles induced by combination PD-1/CTLA-4 blockade and cellular features associated with the development of severe irAEs in treated patients with melanoma. Understanding the features of systemic immune activation and function altered by checkpoint blockade, as well as the specific immune features associated with development of irAE, is critical in developing a more precise prediction of potential effects and consequences of checkpoint inhibitor therapies.

We demonstrated that the frequency of naive T cells, especially naive CD4^+^ T cells, before treatment is associated with the development of a severe irAE in this cohort. These results differ somewhat from those in an observational cohort study of patients with melanoma treated with either PD-1/CTLA-4 blockade or PD-1 blockade reported by Lozano et al. (22), which indicated an association of an increased proportion of effector memory CD4^+^ T cells among total PBMC with development of severe irAEs. However, in the study by Lozano et al., multiple CD4 T cell populations, including naive CD4^+^ T cells, quantified among total PBMC showed the same direction of effect as did naive CD4^+^ T cells in our study. Our study refines the specific CD4^+^ T cell population most associated with severe irAEs, here using analysis of a cohort of patients treated with a uniform ICI therapy in the context of a clinic trial. Although we did not have TCR sequencing in our study, the observation that increased TCR repertoire diversity is also associated with more severe irAEs (22) is conceptually consistent with the findings in our study. An increased number of naive T cells should correspond with an increased overall TCR repertoire diversity and represents a younger or less experienced immune system. This observation is also consistent with a separate prior observational study of 28 patients with melanoma treated with different ICI therapies that also indicated an association of naive T cells at baseline with development of severe irAEs, as well as clinical benefit (21).

Profiling T cells before and after combination PD-1/CTLA-4 therapy demonstrated a large set of prominent changes in circulating T cell phenotypes, including marked expansion of activated, CD38^hi^Ki67^+^ T cells among both CD4^+^ T cells and CD8^+^ T cells. The inclusion of an anti-IgG4 antibody in the staining panel enabled detection and evaluation of cells directly bound by nivolumab. These nivolumab-bound CD4^+^ T cells are highly enriched in activated and cytotoxic CD4^+^ T cell phenotypes, consistent with our prior analyses (27), suggesting that T cells released from PD-1/CTLA-4 inhibition acquire an activated, proliferating phenotype systemically. A recent study corroborates our findings of an expansion of Ki67^+^CD38^hi^ T cells early following ICI therapy and identifies these cells as expanded early after treatment in those with irAEs (31). Importantly, our data do not show a link between frequency of nivolumab-bound T cells and irAE severity.

While some studies indicate that Tregs are depleted in the tumor microenvironment following CTLA-4 blockade, their change in frequency in circulation has been less well characterized, especially following therapy of both anti–CTLA-4 and anti–PD-1 (32). We identified an increase in FOXP3^+^ Tregs following combination PD-1/CTLA-4 blockade, with the expanded Tregs showing increased expression of Treg and activation markers. These changes in Tregs may be due to CTLA-4 blockade, PD-1 blockade, or both. While we did not include an antibody to detect cell-bound ipilimumab in our staining panel, it is plausible that CTLA-4 blockade affects Treg numbers and phenotype. Ipilimumab can induce antibody-dependent cellular cytotoxicity and phagocytosis on intratumoral Tregs (33–37); however, the absolute numbers of intratumoral Tregs increases in melanoma tumors after CTLA-4 blockade (38). Absence of PD-1 on Tregs can also change Treg features and function, including through upregulation of CD30 expression (39). Future work should include gene expression level analysis on Tregs before and after ICI therapy to further characterize changes to individual Tregs populations, such as CD25^hi^ or CD25^lo^ Tregs.

This cohort is made up of patients with a single malignancy treated with 1 specific protocol of combination PD-1/CTLA-4 blockade. While our results cannot be extrapolated to other treatment protocols at this time, our analysis is strengthened by lack of extraneous heterogeneity. Our study is limited by 1 cohort of patients and, thus, should be validated within separate cohorts and methods in the future. Additionally, in this cohort, we cannot assess cellular features associated with a lack of irAE development because almost all of the patients studied developed at least 1 mild irAE. The study was not powered to evaluate organ-specific irAE manifestations, and the multiple irAEs that occur in individual patients complicate testing for cellular associations with individual organ-specific irAE. The cytometric profiling approach focused primarily on lymphocyte features; thus, we have not fully captured the range of changes on myeloid and DC populations. Nonetheless, the use of high-dimensional profiling of longitudinal samples from a clinical trial provide a robust, broad assessment of cellular phenotypes associated with checkpoint blockade in this setting. Our study specifically associates circulating naive CD4^+^ T cells with predisposition to high severity irAE.

## Methods

### Sex as a biological variable.

We included both male and female patient samples in our analysis. We have included sex as a covariate in our statistical analyses since proportions of naive T cells especially CD8^+^ T cells can be associated with age (Supplemental Figure 6, A and B). We have noted this in the corresponding figure legends.

### Mass cytometry of ICI-treated PBMCs.

Cryopreserved PBMCs were thawed, washed, and counted for mass cytometry staining. Two million live PBMCs were stained for mass cytometry from each sample. In total, 86 samples were stained in 5 batches. Additionally, a reference sample isolated from a leukoreduction collar was included in each batch. Antibodies were acquired from Fluidigm and Longwood Medical Area CyTOF Core. Clones of the antibodies can be found in Supplemental Table 2. Cells were stained for viability using 103Rhodium, followed by FC block (Invitrogen). Surface staining was done in cell staining buffer (Fluidigm) for 30 minutes followed by fixing and permeabilization with FoxP3/Transcription Factor Staining Buffer Set (Invitrogen) and barcoding (Fluidigm). Intracellular staining was done on pooled barcoded samples. Following fixing in 4% PFA, cells were diluted in CyTOF PBS containing appropriate concentrations of Intercalator-Ir, washed, and resuspended in cell acquisition plus buffer containing EQ beads (Fluidigm). Cells were acquired on a CyTOF-XT (Fluidigm).

### Mass cytometry data analysis of ICI-treated PBMCs.

Cytometry data were normalized and debarcoded as previously described (40). Live cells were gated using FlowJo (BD) as DNA^+^103Rh^–^Beads^–^ events. Batch correction on total live cells was performed by the cyCombine R package (25). Following batch correction, 15,000 cells per sample were analyzed in an unsupervised manner using the Seurat pipeline for R. Further characterization of CD8^+^ and CD4^+^ T cell populations was carried out by gating for CD3^+^CD8^+^CD4^–^ and CD3^+^CD8-CD4^+^ cells, respectively, followed by unsupervised analysis using the Seurat R package. CD4^+^ and CD8^+^ cluster resolutions were chosen based on ability to identify clear definitions of CD4^+^ or CD8^+^ T cell subsets and then confirmed using the ClusTree package. Analysis by biaxial gating was done on live batch corrected cells using FlowJo (BD).

### Flow cytometry staining and analysis of ICI-treated PBMCs.

Cryopreserved PBMCs were thawed, counted, and washed as before. Approximately 1 million PBMCs were stained for each of 86 samples in 5 batches. Antibodies included anti-CD14 134620 AF350 (R&D Biosystems, FAB3832U), anti-CD19 HIB19 APC-Cy7 (BioLegend, 302218), anti-CD20 2H7 BV605 (302334), anti-CD27 M-T271 BV421 (BioLegend, 356418), anti- CD38 HIT2 BV786 (BioLegend, 303530), anti-CD21 Bu32 APC (BioLegend, 354906), anti-PE Bu15 CD11C (BioLegend, 337206), anti-IgD Ia6-2 Percp-cy5.5 (BioLegend, 348208), anti-IgG M1310G05 BV510 (BioLegend, 410716), anti-IgA IS11-8E10 FITC (Miltenyi, 130-113-481), anti-IgM MHM-88 PE-Cy7 (BioLegend, 314532), CD24 ML5 BV711 (BioLegend, 311136), anti-BDCA2 CD303 BUV737 (BD Biosciences, 748414), and anti-CD123 6H6 BV650 (BioLegend, 306020). Analysis by biaxial gating was performed using FlowJo (BD).

### Statistics.

Comparisons of frequencies of clusters of cells before and after therapy was done using Wilcoxon paired test in GraphPad Prism. irAE severity was considered high if greater than or equal to a max grade of 3. Comparisons of frequencies of cells between high severity and low severity irAE patients was done with a Mann-Whitney *U* test in GraphPad Prism.

To identify cell clusters associated with high severity irAEs at the single cell level, we utilized MASC as previously described (41). To identify cells belonging to neighborhoods associated with high severity irAEs, we utilized CNA also as previously described (28). In both types of analysis, cells or clusters were considered significantly associated if they had an adjusted *P* value of less than 0.1. ROC analysis was completed using the R package pROC and function glm. Analysis was completed for T cell cluster proportions and T cell proportions identified by bi-axial gating.

### Study approval.

Patient research was performed in accordance with the IRB at Memorial Sloan Kettering and Mass General Brigham. Blood samples were obtained through the ADAPT-IT from patients with melanoma before and after receiving 2 doses of both nivolumab and ipilimumab (5). Demographics of each patient can be found in Supplemental Table 1. Mononuclear cells from PBMCs were isolated by density centrifugation using Ficoll-Paque Plus (GE healthcare) and cryopreserved in 10% DMSO in FBS and stored in liquid nitrogen.

### Data availability.

Values for all data points in graphs are reported in the Supporting Data Values file. R codes for analysis will be made available upon request.

## Author contributions

DAR, ARB, LD, and KEM conceptualized the project. KEM, ME, and IAA performed experiments. KEM analyzed data. AH and NG advised data analysis. MP provided patient samples. KEM and DAR drafted the manuscript, and all authors edited the manuscript.

## Funding support

This work is the result of NIH funding, in whole or in part, and is subject to the NIH Public Access Policy. Through acceptance of this federal funding, the NIH has been given a right to make the work publicly available in PubMed Central.

- Innovative Research Award from the Rheumatology Research Foundation (to DAR, ARB, LD)
- Career Award in Medical Sciences from the Burroughs Wellcome Fund (to DAR)
- NIAMS P30 AR070253
- National Cancer Institute/National Institutes of Health Cancer Center Support Grant (P30CA008748) (to MP).

## Supplementary Material

Supplemental data

ICMJE disclosure forms

Supplemental table 1

Supporting data values

## Figures and Tables

**Table 1:**
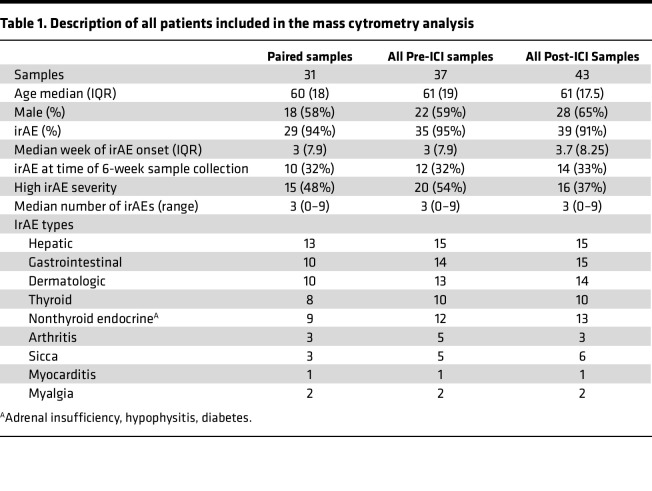
Description of all patients included in the mass cytrometry analysis

## Version 1 11/26/2025

In-Press Preview

## Version 2 01/23/2026

Electronic publication
