# Supplementary material for: Pretreatment naive T cells are associated with severe irAE following PD-1/CTLA-4 checkpoint blockade for melanoma
Source: JCI Insight. 2025 Nov 25;11(2):e198203. doi: 10.1172/jci.insight.198203 (PMC12892909; doi:10.1172/jci.insight.198203)
Supplement: Supplemental data [file jciinsight-11-198203-s196.pdf]

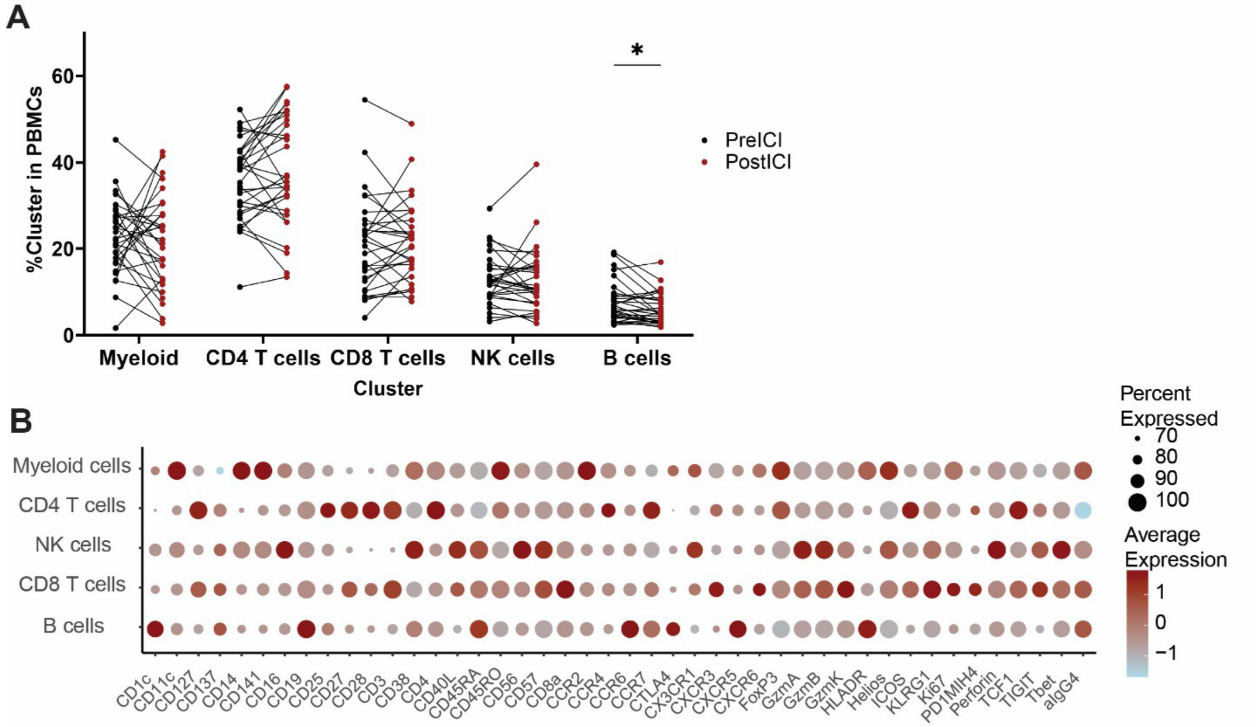

Supplemental Figure 1

A) Paired analysis of frequency of major cell clusters before vs after treatment per patient. * $p<0.05$ by Wilcoxon paired test. B) DotPlot of all mass cytometry markers in PBMCs by low resolution clusters (see Figure 1B-D).

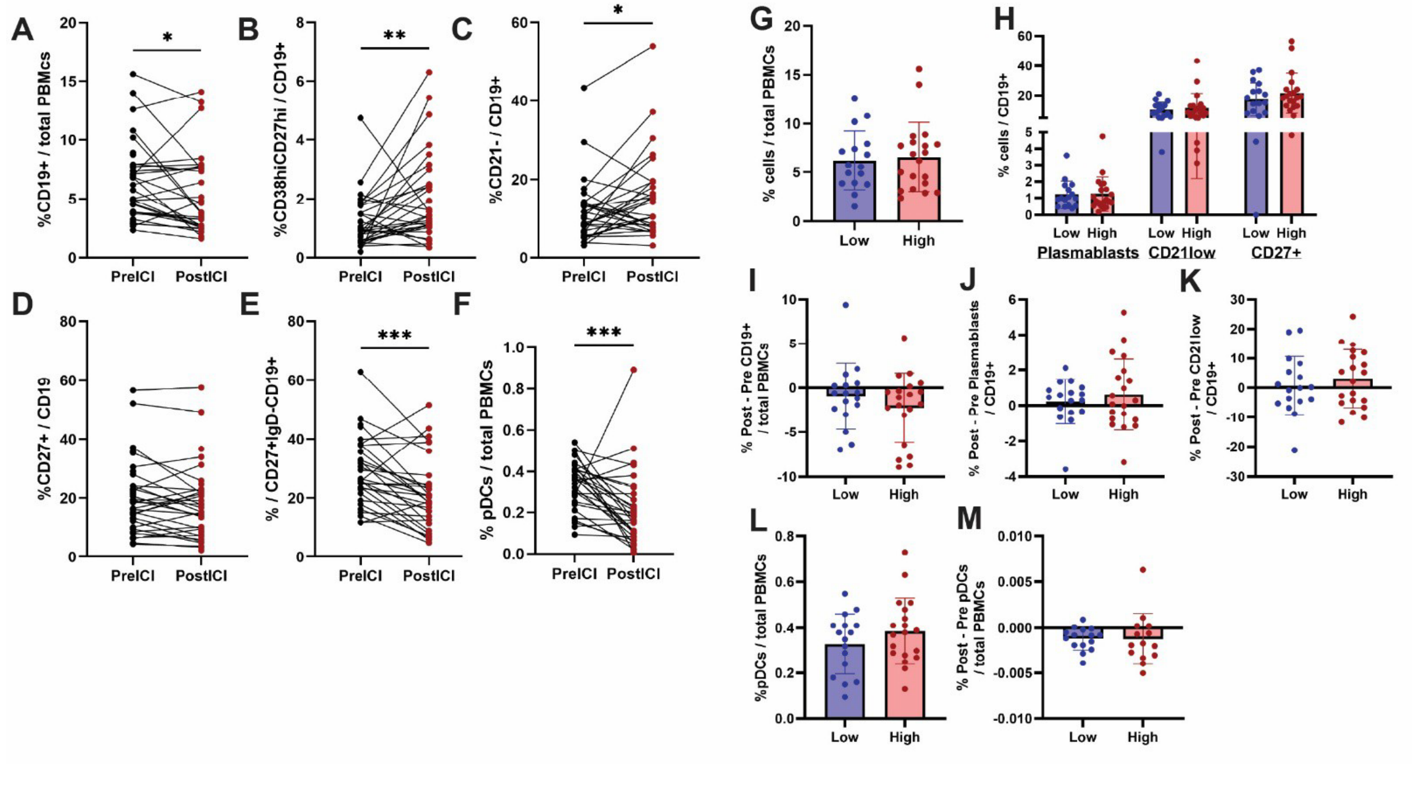

Supplemental Figure 2. Changes to B cell frequencies following ICI treatment. A - E) Paired analysis of frequency of indicated B cell markers analyzed by flow cytometry before vs after treatment per patient in

indicated populations. F) Paired analysis of frequency of plasmacytoid dendritic cells (gated as CD3CD19-CD14-CD11C-BDCA2+CD123+) before vs after treatment per patient. A G, H) Analysis of frequency of indicated B cell populations analyzed by flow cytometry before treatment per patient in indicated populations. Frequencies of B cells in patients with low and high severity irAEs are compared. IK) Change in frequency of B cell populations after - before treatment. L) Frequency of plasmacytoid dendritic cells (gated as CD3-CD19-CD14-CD11C-BDCA2+CD123+). M) Change in frequency of plasmacytoid dendritic cells after - before treatment. Red bars indicate patients with high severity irAEs, and blue bars indicate patients with low severity irAEs. -F: * $p<0.05$, ** $p < 0.01$, *** $p <0.001$ by Wilcoxon paired test. G-M: All plots are not significant by Mann Whitney test.

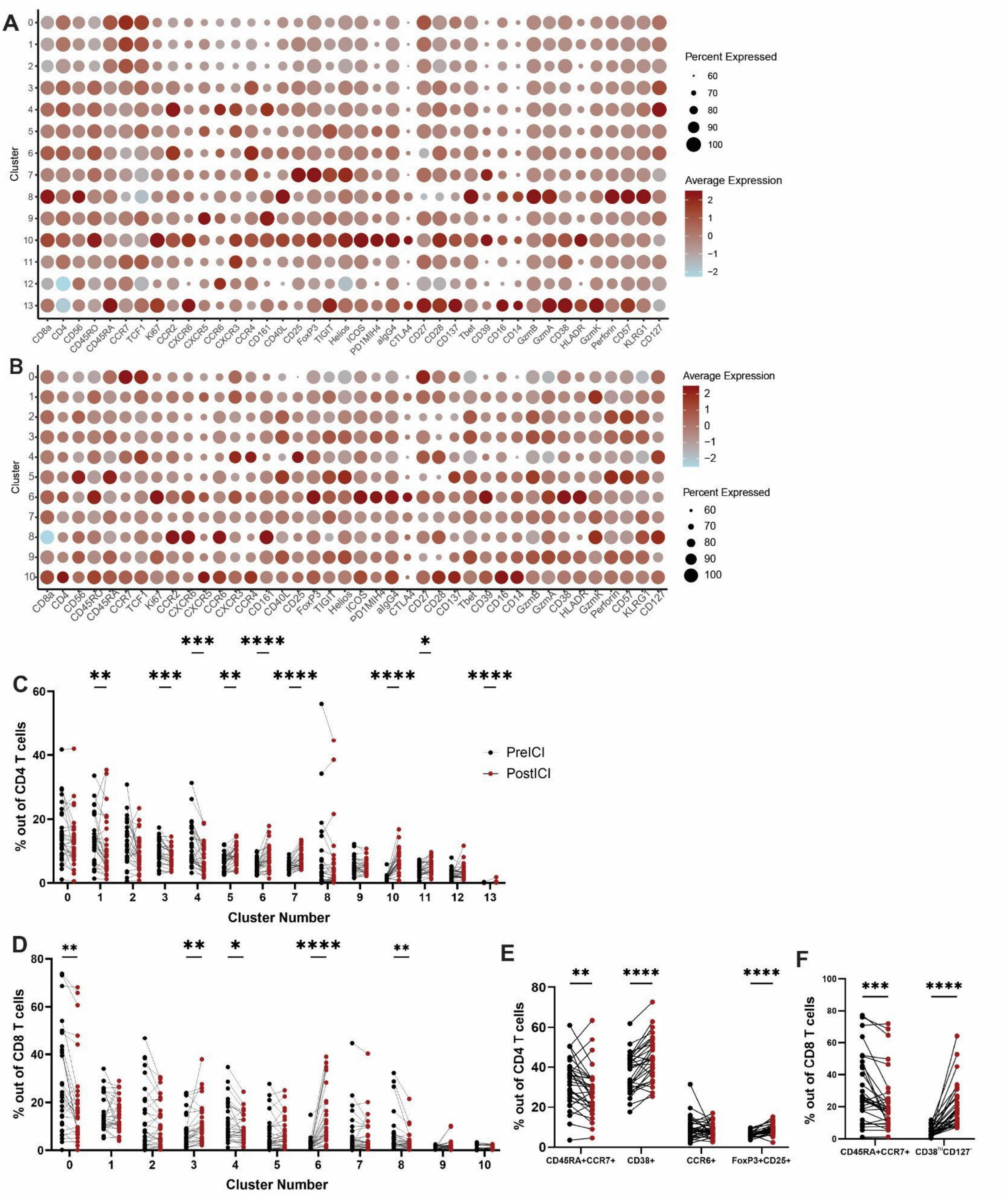
A
Cluster
0
1
2
3
4
5
6
7
8
9
10
11
12
13
CD8a CD4 CD56 CD45RO CD45RA CCR7 TCF1 Ki67 CCR2 CXCR6 CXCR5 CCR6 CXCR3 CCR4 CD161 CD40L CD25 FoxP3 TIGIT Helios ICOS PD1MIH4 aIgG4 CTLA4 CD27 CD28 CD137 Tbet CD39 CD16 CD14 GzmB GzmA CD38 HLADR GzmK Perforin CD57 KLRG1 CD127
Percent Expressed
60
70
80
90
100
Average Expression
2
1
0
−1
−2
B
Cluster
0
1
2
3
4
5
6
7
8
9
10
CD8a CD4 CD56 CD45RO CD45RA CCR7 TCF1 Ki67 CCR2 CXCR6 CXCR5 CCR6 CXCR3 CCR4 CD161 CD40L CD25 FoxP3 TIGIT Helios ICOS PD1MIH4 aIgG4 CTLA4 CD27 CD28 CD137 Tbet CD39 CD16 CD14 GzmB GzmA CD38 HLADR GzmK Perforin CD57 KLRG1 CD127
Average Expression
2
1
0
−1
−2
Percent Expressed
60
70
80
90
100
C
% out of CD4 T cells
60
40
20
0
0 1 2 3 4 5 6 7 8 9 10 11 12 13
Cluster Number
PreICI
PostICI
** *** *** ** **** **** **** * ****
D
% out of CD8 T cells
80
60
40
20
0
0 1 2 3 4 5 6 7 8 9 10
Cluster Number
** ** * **** **
E
% out of CD4 T cells
80
60
40
20
0
CD45RA+CCR7+
CD38+
CCR6+
FoxP3+CD25+
** **** ****
F
% out of CD8 T cells
100
80
60
40
20
0
CD45RA+CCR7+
CD38hiCD127−
*** ****

Supplemental Figure 3

A) DotPlot representation of all mass cytometry markers in CD4+ T cells subset from PBMCs (see Figure 2A-C). B) Dot Plot representation of all mass cytometry markers in CD8+ T cells subset from PBMCs (see Figure 2D-F). C) Paired analysis of frequency of all CD4 T cell and D) CD8 T cell clusters before vs after treatment per patient. test. E) Paired analysis of CD4 and F) CD8 T cells analyzed by biaxial gating. * p<0.05, **p<0.01, ***p<0.001, ****p<0.0001 by Wilcoxon paired analysis.

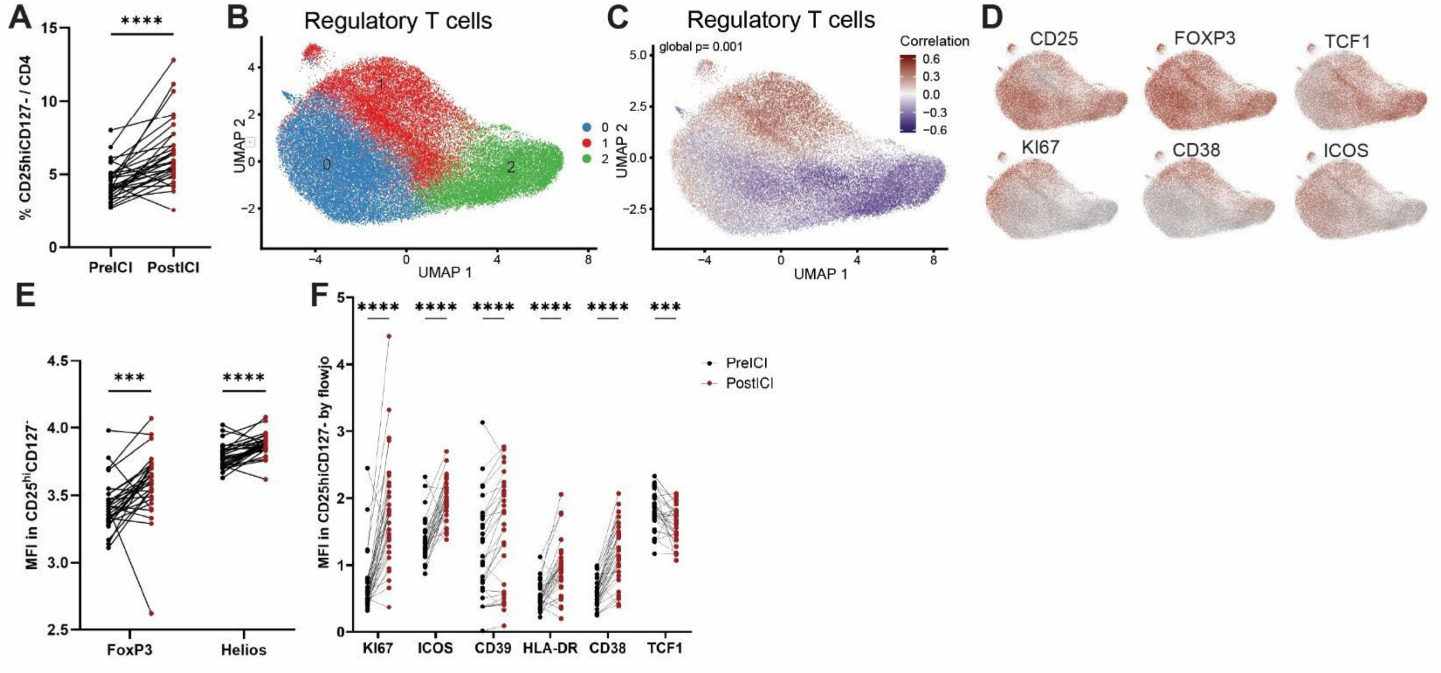

Supplemental Figure 4 A) Paired analysis of frequency of CD25^{hi}CD127^{-} CD4 T cells before and after ICI therapy by biaxial gating. B) UMAP representation following unsupervised clustering of CD4 regulatory T cells. C) CNA analysis of before treatment samples vs after treatment samples. Global p value = 0.001. D) Feature plots of the indicated markers on the Treg UMAP. E) Paired analysis of Median Fluorescent Intensity of FoxP3 and Helios or F) Ki67, ICOS, CD39, HLA-DR, and CD38 in CD25^{hi}CD127^{-} CD4 T cells before and after ICI therapy. ***p<0.001, ****p<0.0001 by Wilcoxon paired analysis.

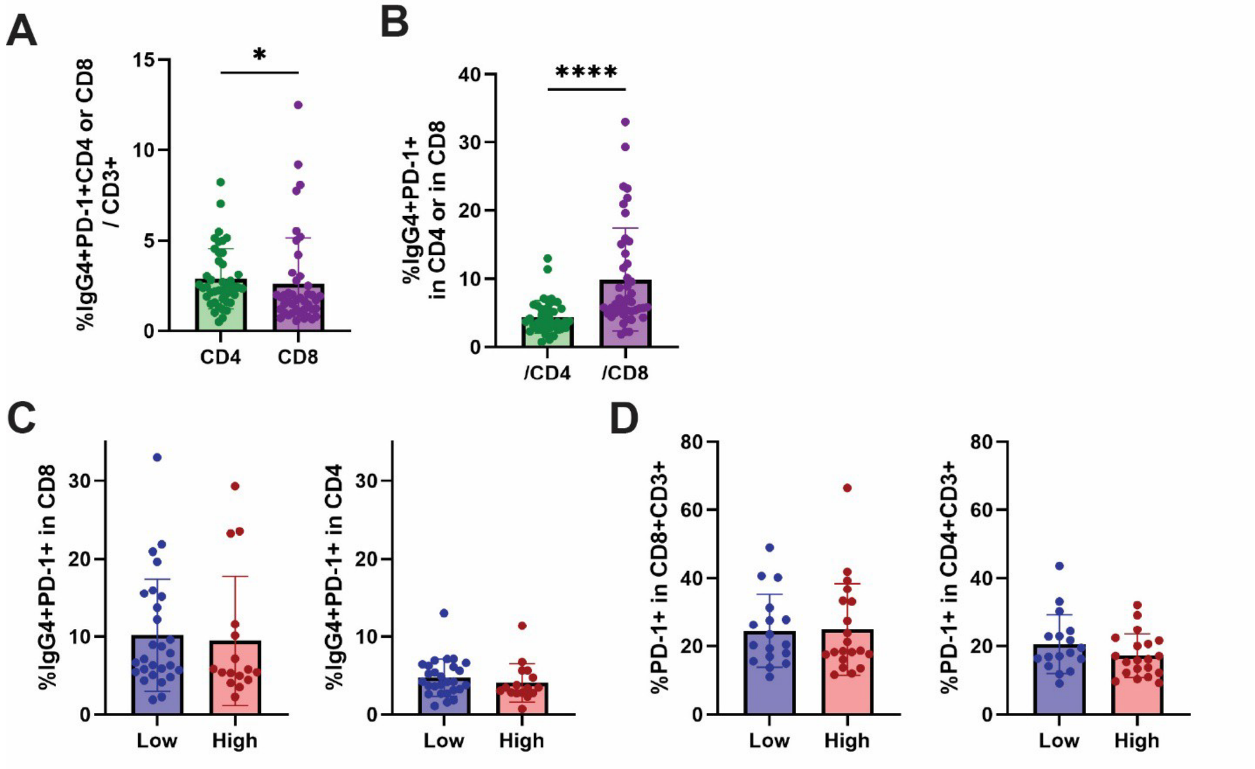

Supplemental Figure 5

A) Frequency of IgG4⁺PD-1⁺ CD4⁺ or CD8⁺ T cells out of CD3⁺ T cells by gating of mass cytometry analysis in the post-treatment samples. B) Frequency of IgG4⁺PD-1⁺ out of CD4⁺ or CD8⁺ T cells by gating of mass cytometry analysis in the post-treatment samples. C) Frequency of IgG4⁺PD-1⁺ out of CD4⁺ (right) or CD8⁺ (left) T cells in patients with low or high severity of irAE by gating of mass cytometry analysis. D) Frequency of total PD-1⁺ out of CD4⁺ (right) or CD8⁺ (left) T cells in pre-treatment samples from patients with low or high severity of irAE by gating of mass cytometry analysis.

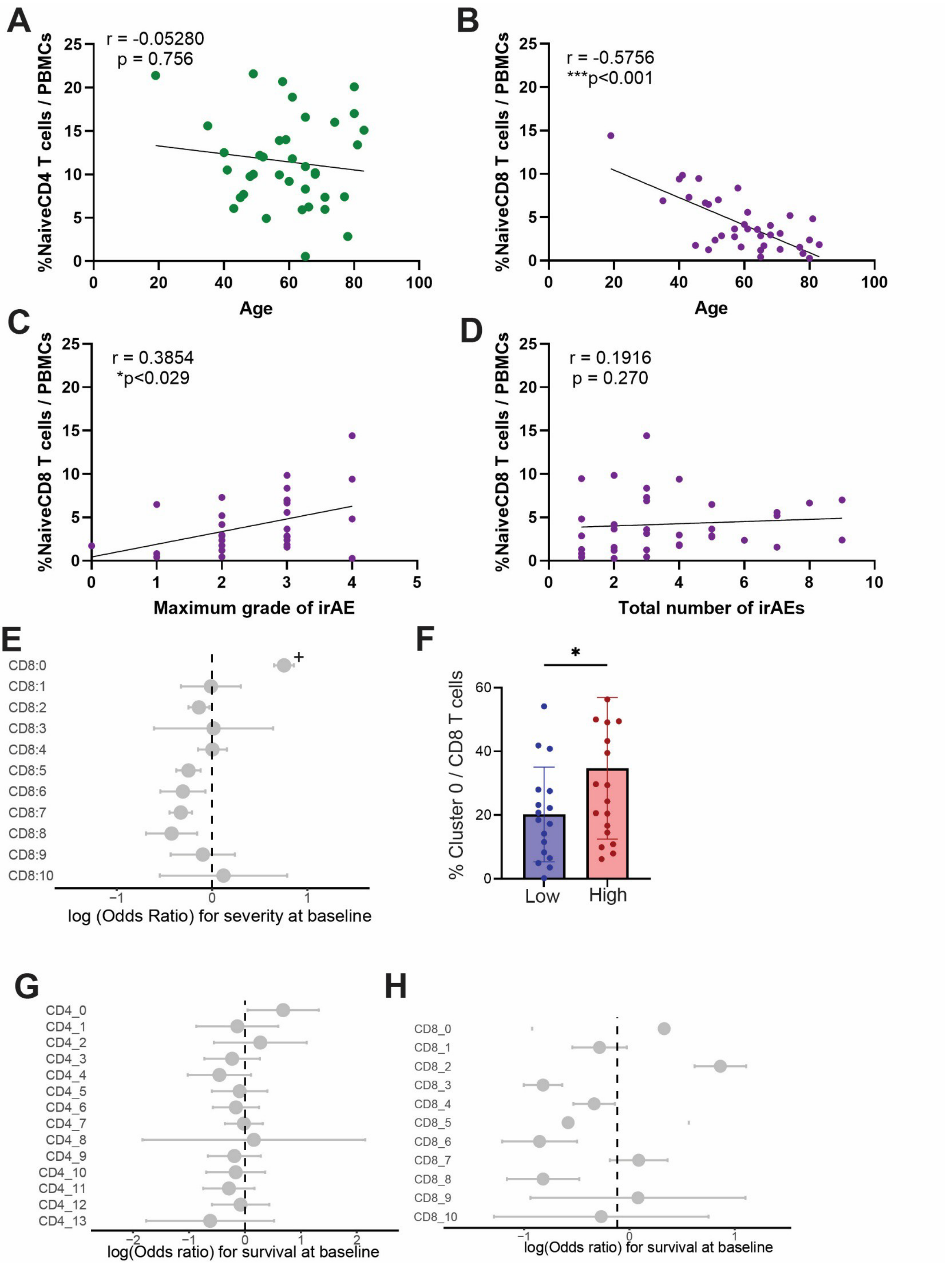
A
r = -0.05280
p = 0.756
%NaiveCD4 T cells / PBMCs
Age
B
r = -0.5756
***p<0.001
%NaiveCD8 T cells / PBMCs
Age
C
r = 0.3854
*p<0.029
%NaiveCD8 T cells / PBMCs
Maximum grade of irAE
D
r = 0.1916
p = 0.270
%NaiveCD8 T cells / PBMCs
Total number of irAEs
E
CD8:0
CD8:1
CD8:2
CD8:3
CD8:4
CD8:5
CD8:6
CD8:7
CD8:8
CD8:9
CD8:10
log (Odds Ratio) for severity at baseline
F
% Cluster 0 / CD8 T cells
Low
High
G
CD4_0
CD4_1
CD4_2
CD4_3
CD4_4
CD4_5
CD4_6
CD4_7
CD4_8
CD4_9
CD4_10
CD4_11
CD4_12
CD4_13
log(Odds ratio) for survival at baseline
H
CD8_0
CD8_1
CD8_2
CD8_3
CD8_4
CD8_5
CD8_6
CD8_7
CD8_8
CD8_9
CD8_10
log(Odds ratio) for survival at baseline

Supplemental Figure 6

A) Spearman correlation of proportion of gated naïve CD4+ T cells ($CD19^{-}CD14^{-}CD3^{+}CD4^{+}CD45RA^{+}CCR7^{+}$) with age. B) Spearman correlation of proportion of gated naïve CD8+ T cells ($CD19^{-}CD14^{-}CD3^{+}CD8^{+}CD45RA^{+}CCR7^{+}$) with age, C) maximum grade or irAE, and D) total number of irAEs diagnosed. E) MASC analysis of CD8 T cell clusters (see Figure 2). + indicates $p < .05$, adj $p = 0.5$. F) Proportion of cluster 0 out of CD8 T cells. $p<0.05$ by Mann Whitney test. G) MASC analysis for overall survival in $CD4^{+}$ and H) $CD8^{+}$ T cells before treatment (survived n = 5, not survived n = 29).
